# Surgical reconstruction of caustic esophageal strictures: a retrospective comparative study of esophagogastroplasty and esophagocoloplasty

**DOI:** 10.1186/s12893-025-03084-4

**Published:** 2025-10-22

**Authors:** Marcos Paulo Ferreira Corrêa Alves Reis, Luiz Ronaldo Alberti, Tarcisio Versiani Azevedo Filho, Adebal de Andrade Filho, Paulo Roberto Lima Carreiro, Bárbara Campos Mattos, Gabriela Souza Fernandes, Isabela de Paula Nogueira Pereira

**Affiliations:** 1 Hospital João XXIII– FHEMIG, Belo Horizonte, MG Brazil; 2 Universidade Federal de Minas Gerais, UFMG, Belo Horizonte, MG Brazil; 3 Hospital Júlia Kubitschek– FHEMIG, Belo Horizonte, MG Brazil; 4 Department of Surgery, Faculdade de Ciências Médicas de Minas Gerais, Belo Horizonte, Brasil

**Keywords:** Esophageal Stenosis, Esophagectomy, Sodium Hydroxide, Trauma

## Abstract

**Background:**

Caustic soda ingestion can result in severe esophageal injuries, leading to chronic strictures that may require surgical reconstruction. Esophagogastroplasty and esophagocoloplasty are the most common surgical options; however, comparative outcome data remain scarce.

**Methods:**

We conducted a retrospective analysis of medical records from patients treated for caustic soda ingestion at Hospital João XXIII (HJXXIII), Belo Horizonte, Brazil, between 2014 and 2020. Data were reviewed and finalized in 2024. Among 277 patients, 101 adults had acute esophageal injuries classified by esophagogastroduodenoscopy (EGD). Forty-seven developed esophageal strictures, of whom 34 did not respond to endoscopic dilation and required surgical reconstruction. Of these, 19 underwent esophagogastroplasty and 15 underwent esophagocoloplasty due to significant gastric injuries (atrophy, severe adhesions, or previous gastrostomy).

**Results:**

Esophagogastroplasty was performed in 55.9% and esophagocoloplasty in 44.1% of surgical cases. The postoperative mortality rate was 0% in the esophagogastroplasty group and 20% (3/15) in the esophagocoloplasty group. Anastomotic leaks occurred in 21.1% (4/19) and 53.3% (8/15), respectively (*p* = 0.058, trend toward significance). Most patients resumed oral feeding, and only one required postoperative dilation for anastomotic stricture.

**Conclusions:**

Esophagogastroplasty was associated with lower rates of major postoperative complications and no mortality in this cohort of chronic caustic esophageal strictures. Further multicenter studies are needed to confirm these findings and guide surgical decision-making.

## Introduction

Caustic lesions of the upper gastrointestinal tract remain among the most challenging conditions faced by gastroenterologists and surgeons [1]. According to Harlak et al., esophageal stenosis is the main long-term complication of caustic soda ingestion, and only a small percentage of adult patients undergo surgical treatment [2]. However, the best surgical method to manage severe corrosive stenosis remains debated [2].

In American teenagers, 12% to 20% of caustic soda ingestions are due to suicide attempts, which are more frequent among females [3]. Among adults, most cases are voluntary, usually with suicidal intent [3, 4]. Psychiatric disorders are often associated, especially in women, while alcoholism is cited as a cause for accidental ingestion [3].

Statistical data are scarce in Brazil; however, the incidence is believed to be similar to that in the USA [3]. Caustic lesions occur when substances with a pH < 2 or > 12 are ingested [1]. The esophagus and stomach are the most affected regions, as the substance often remains there longer. However, injuries can occur anywhere in contact with the substance, including the oral mucosa, pharynx, upper airway, and duodenum [5].

Despite the 2016 esophageal dilation guidelines, therapeutic response, optimal timing, and dilation intervals vary, and there is no strong consensus in the literature [6]. When multiple dilations fail or when patients refuse repeated procedures, more aggressive techniques to reconstruct the tract from the hypopharynx to the stomach are indicated [7].

Most caustic soda ingestions do not require surgical intervention [8]. Surgical treatment is divided into emergency procedures and late reconstructions [7]. Surgical centers specializing in upper gastrointestinal tract (GIT) surgery, especially in developed countries, report only a small number of these cases per year [9]. Consequently, knowledge about the best approach remains limited [9].

In Brazil, caustic soda ingestion remains a significant concern due to its severity [3]. Its easy access in household products leads to frequent accidental or intentional ingestions [3]. Most research focuses on pediatric patients, who account for up to 80% of accidental ingestions [7, 9]. Although patients with esophageal caustic lesions are at risk for strictures, optimal management and treatment choices are still debated [10].

Despite the connection between the endoscopic grade of the caustic lesion and the risk of developing esophageal stenosis, few studies have explored the association with the type of agent ingested, mainly because each agent leads to different pathophysiological mechanisms [5, 11].

Therapeutic EGD in patients with caustic soda-induced strictures has a low success rate. Approximately 50% to 70% of these patients ultimately require surgery. The literature on these injuries in adults is scarce and even more limited and controversial regarding surgical approaches [8, 12]. Therefore, a comparative study between esophagogastroplasty and esophagocoloplasty in patients with caustic soda-induced strictures is warranted.

We aimed to describe and perform a comparative analysis of complications in patients undergoing esophagectomy with esophagogastroplasty versus esophagocoloplasty for caustic soda-induced strictures.

## Detail of the methodology

### Study design and patient selection

We conducted an active search of medical records from patients treated for caustic soda ingestion at Hospital João XXIII (HJXXIII) in Belo Horizonte, Brazil, between 2014 and 2020. We reviewed 277 records and included 101 adult patients with acute esophageal injuries classified by esophagogastroduodenoscopy (EGD). The review and analysis were completed in 2024 to ensure updated interpretation. We excluded patients younger than 15 years, those with acidic injuries, those lost to outpatient follow-up, patients without esophageal injuries, and those not classified by EGD. The Zargar classification was used to grade injuries from grade 0 (normal mucosa) to grade IIIb (extensive necrosis and perforation). We defined refractory stricture as a stricture not responsive to endoscopic dilation or technically impossible to dilate.

**Fig. 1:**
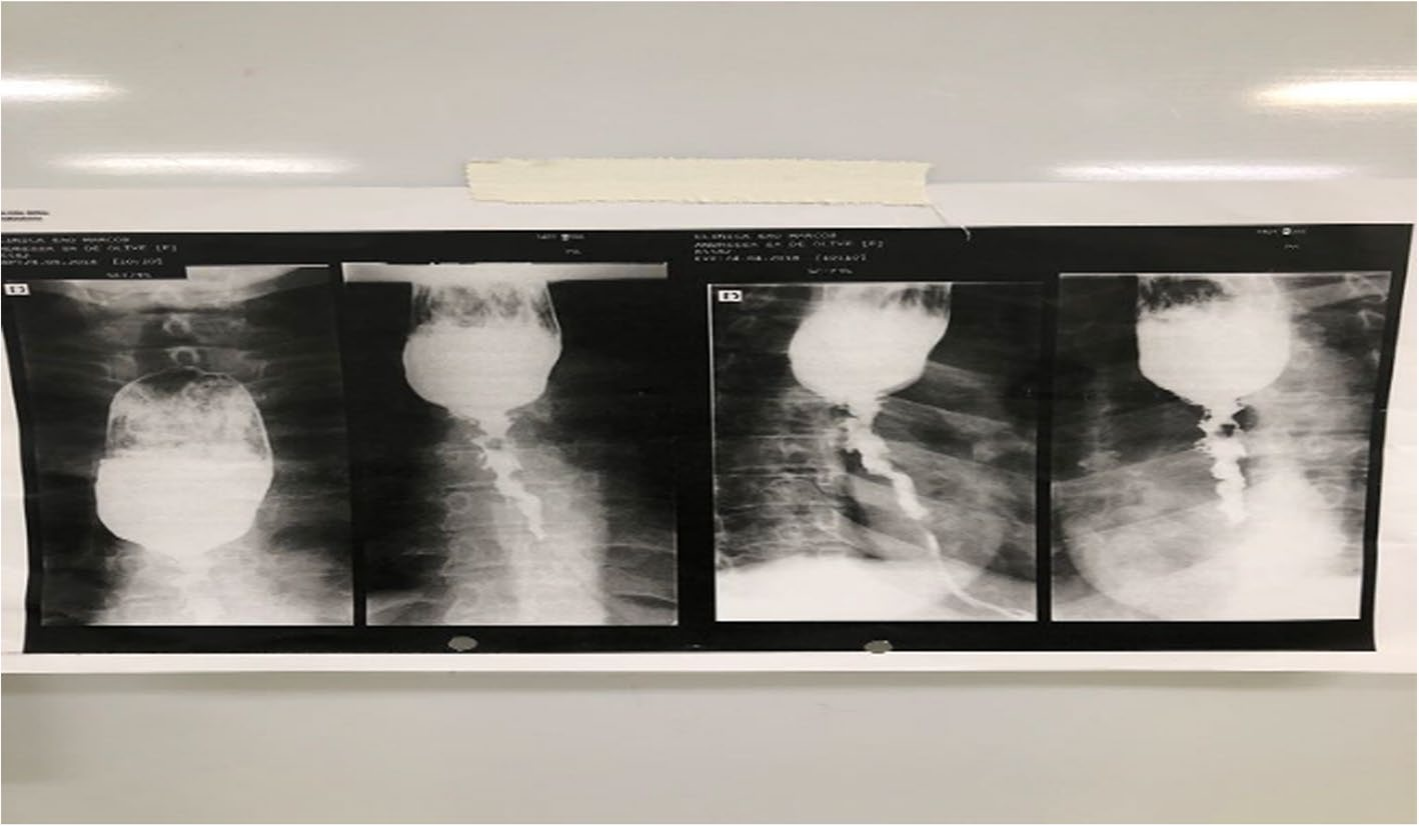
Barium esophagogram showing middle and distal esophageal stenosis

After discharge, patients were followed at the General Surgery outpatient clinic of HJXXIII and referred to Hospital Júlia Kubitschek (HJK) in cases of chronic complications. We evaluated the prevalence and factors associated with esophageal strictures, determined by the presence of dysphagia, sialorrhea, weight loss, or imaging abnormalities (Fig. 1).

Of the 101 patients who had follow-up at HJK, 47 developed esophageal strictures. Among these, 34 did not respond to endoscopic dilation and required surgical reconstruction. Nineteen patients underwent esophagogastroplasty, while 15 with significant gastric injuries—such as gastric atrophy, severe adhesions, or previous gastrostomy—that precluded using the stomach for reconstruction (criteria assessed intraoperatively by the attending surgeon) underwent esophagocoloplasty. We collected and compared baseline characteristics, including age, sex, comorbidities, stricture severity, and time to surgery, to evaluate potential selection bias.

This study adhered to the STROBE guidelines for observational studies. We present a comprehensive summary of patient inclusion, exclusion, and surgical allocation in the STROBE summary table. We included patients with refractory strictures who did not respond to endoscopic dilation or had complete strictures precluding guidewire passage. We excluded patients with severe comorbidities precluding surgery, incomplete records, or those lost to follow-up. We addressed missing data by reviewing medical records and, when necessary, contacting treating physicians. We chose a retrospective design due to the rarity and urgency of the condition, recognizing its inherent limitations.

We retrospectively reviewed follow-up data to assess long-term functional outcomes, including resumption of oral feeding, need for postoperative dilations, and nutritional status. However, no standardized complication grading system (e.g., Clavien-Dindo) was uniformly applied during the study period Table 1.

**Table 1:** STROBE patient flow summary Table

Section	Details
Initial population	277 patients with caustic ingestion (2014–2020)
Screening and inclusion criteria	101 adult patients with acute esophageal injuries, evaluated by esophagogastroduodenoscopy (EGD) within 72 h after ingestion, classified using the Zargar classification
Exclusion criteria	• Age < 15 years • Acidic injuries • No esophageal injuries • Not evaluated by EGD • Lost to follow-up
Development of esophageal stricture	47 patients developed chronic esophageal strictures
Management of strictures	• 13 patients responded to endoscopic dilation (not included in surgical cohort) • 34 patients did not respond to endoscopic dilation → indicated for surgery
Surgical reconstruction allocation	• 19 patients underwent esophagogastroplasty • 15 patients underwent esophagocoloplasty (due to significant gastric injuries, including gastric atrophy, severe adhesions, or previous gastrostomy)

### Esophagogastroplasty

**Fig. 2:**
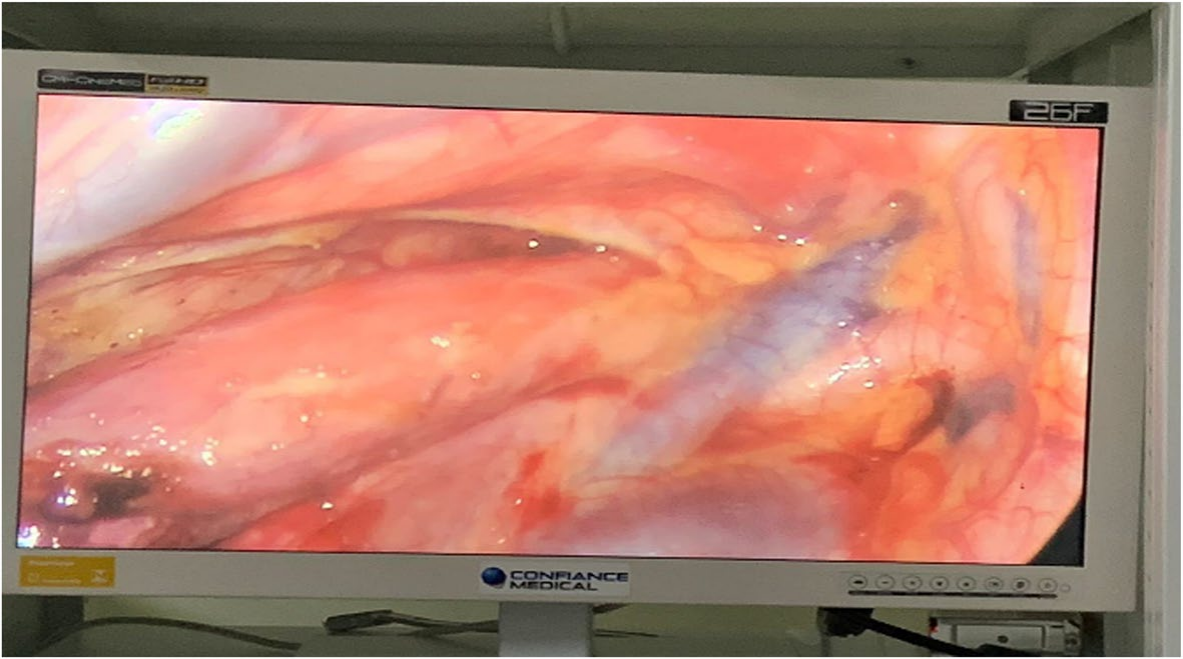
Thoracic esophagus dissected in videothoracoscopy

We performed esophagogastroplasty using video-assisted thoracotomy on the right side, with the patient in the supine position under general anesthesia. We dissected the esophagus with coagulator forceps in the posterior mediastinum, which was often hampered by caustic periesophagitis (Fig. 2).

**Fig. 3:**
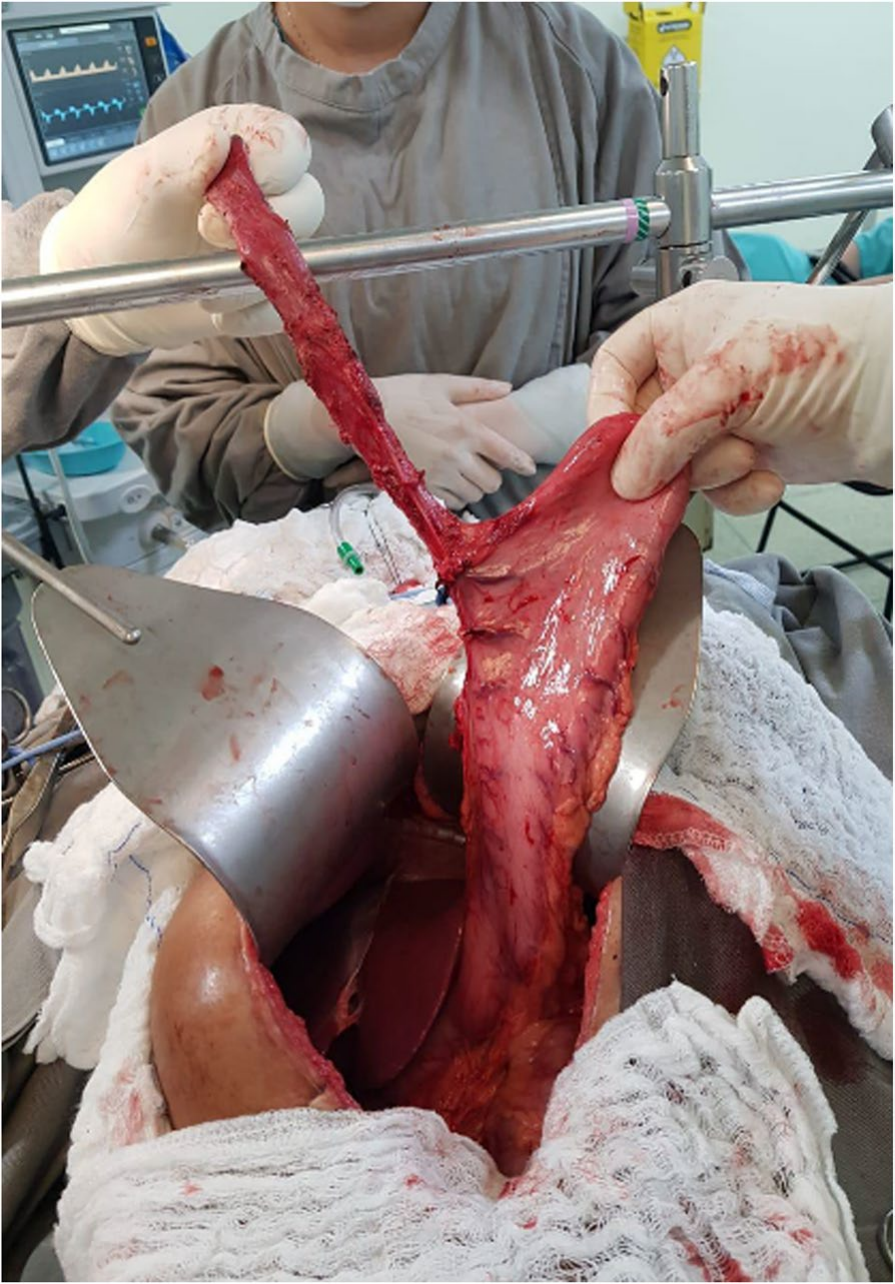
Stomach accessed by laparotomy

**Fig. 4:**
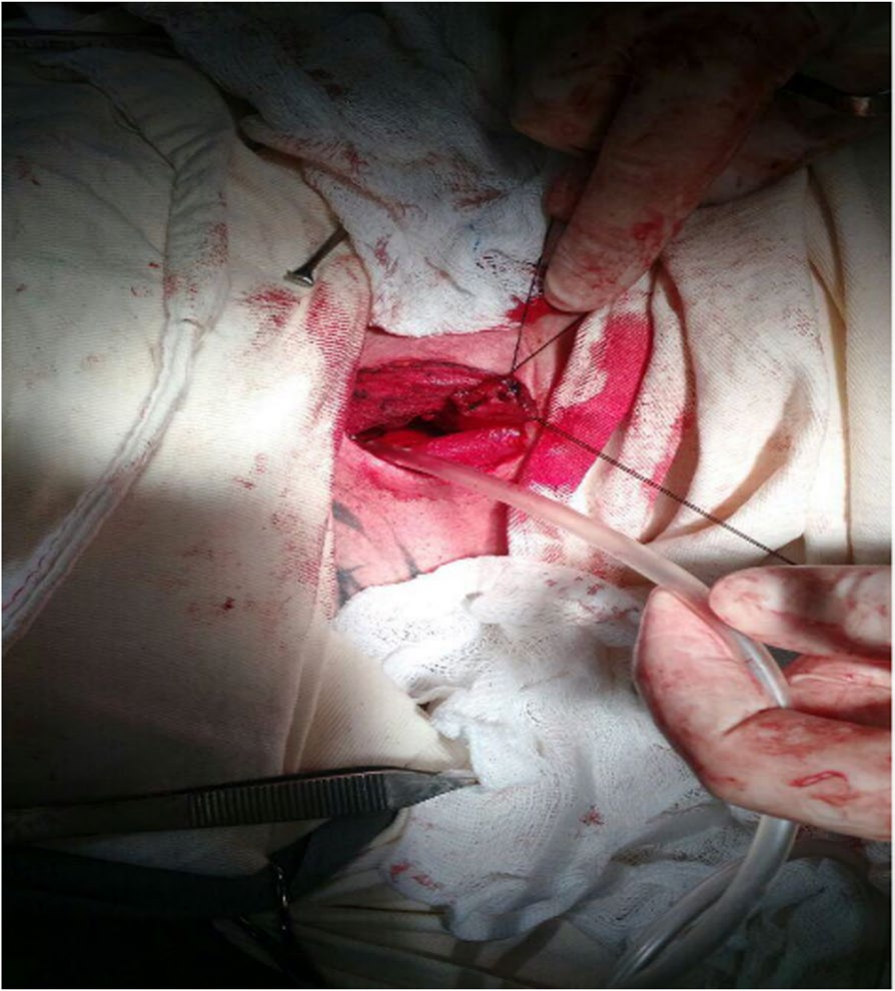
Cervical esophagus

We simultaneously prepared the gastric tube via laparotomy and the cervical esophagus via left cervicotomy, with the patient in the dorsal horizontal position (Figs. 3 and Fig. 4).

**Fig. 5:**
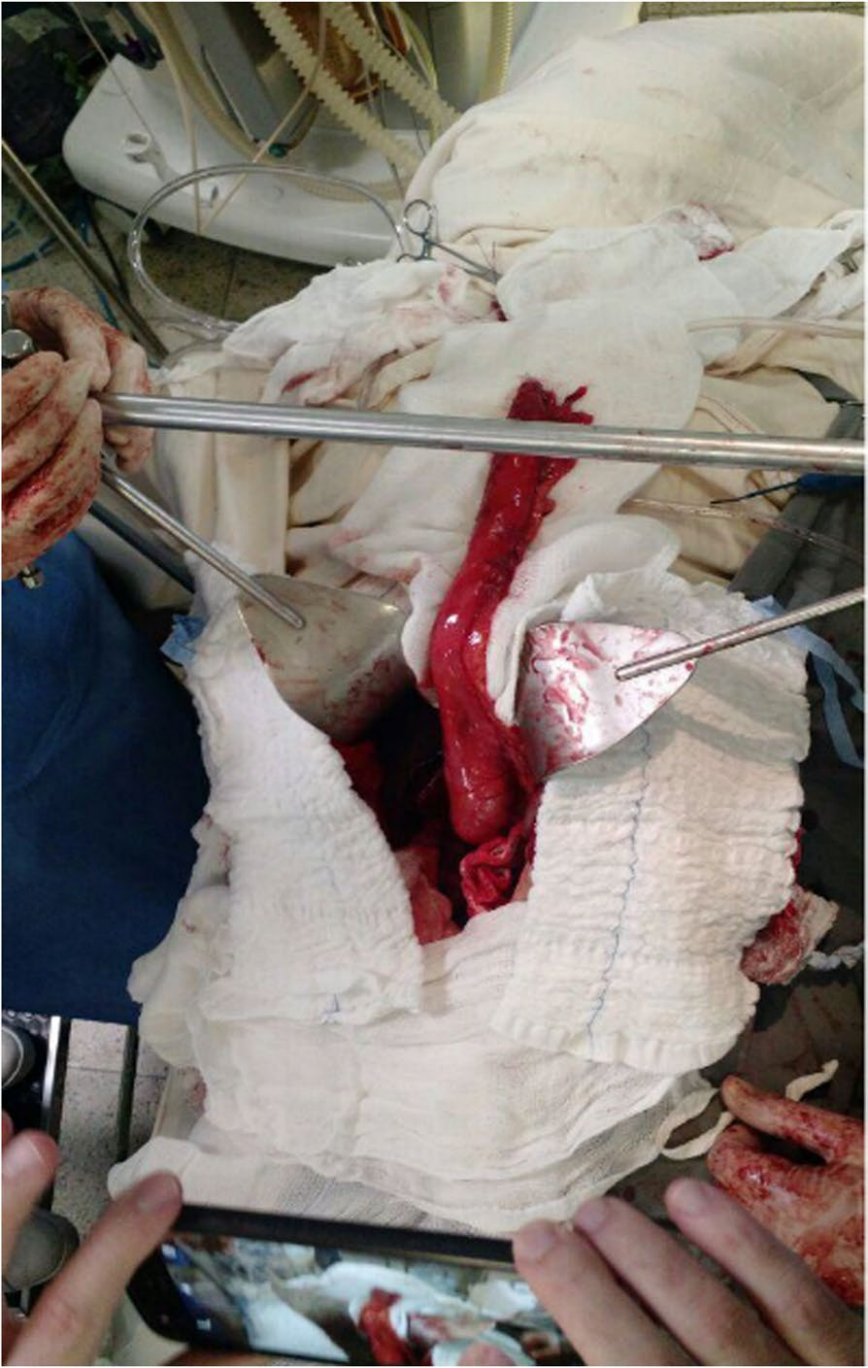
Gastric tube

**Fig. 6:**
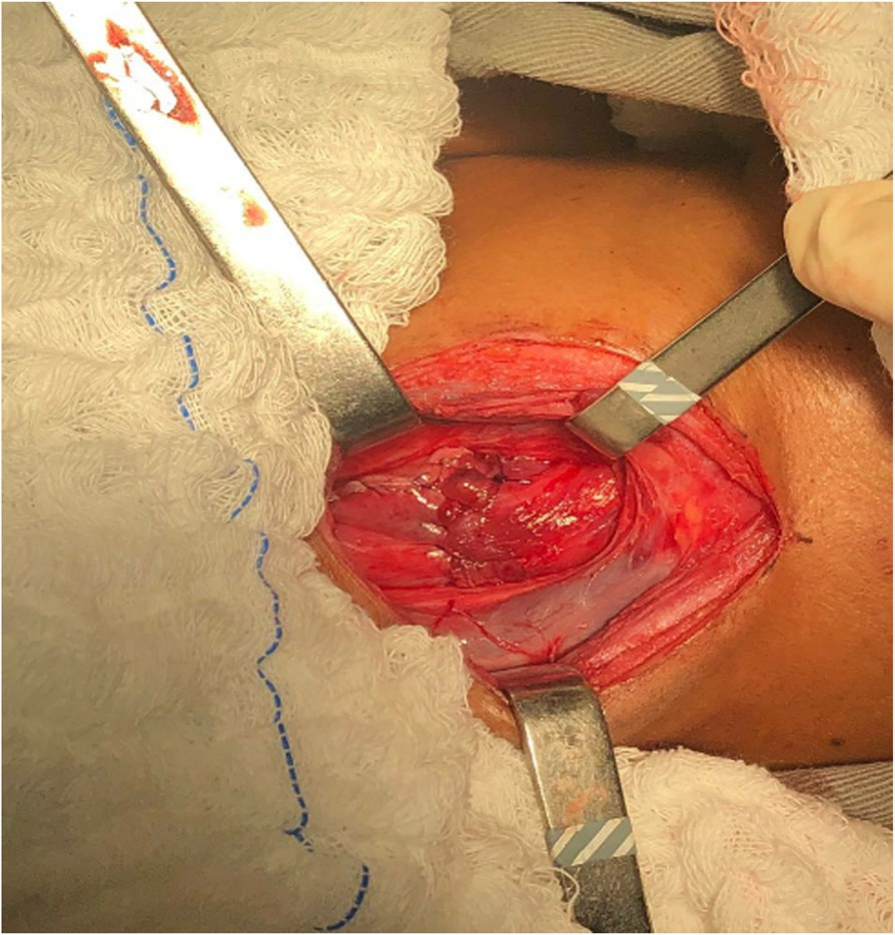
Cervical esophagogastric anastomosis

We prepared the cervical esophagus after regional dissection and sectioned it to assess anastomosis feasibility. We passed a nasogastric tube from the neck to the abdomen, allowing the gastric tube to be retracted to the cervical region after division. We performed the esophagogastric anastomosis manually, using either single or two-layer techniques (separate or continuous), depending on the surgeon’s preference Figs. 5 and Fig. 6).

### Esophagocoloplasty

**Fig. 7:**
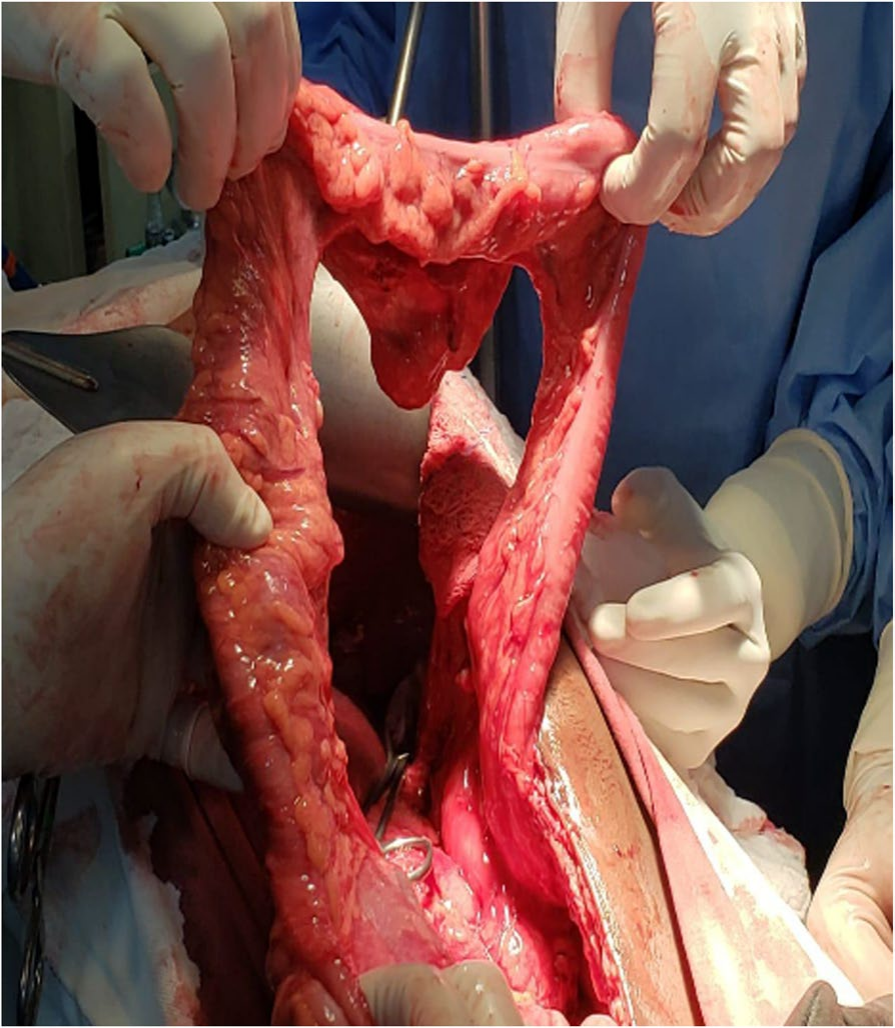
Preparation of the colonic loop; Ligation of middle colic vessels

**Fig. 8:**
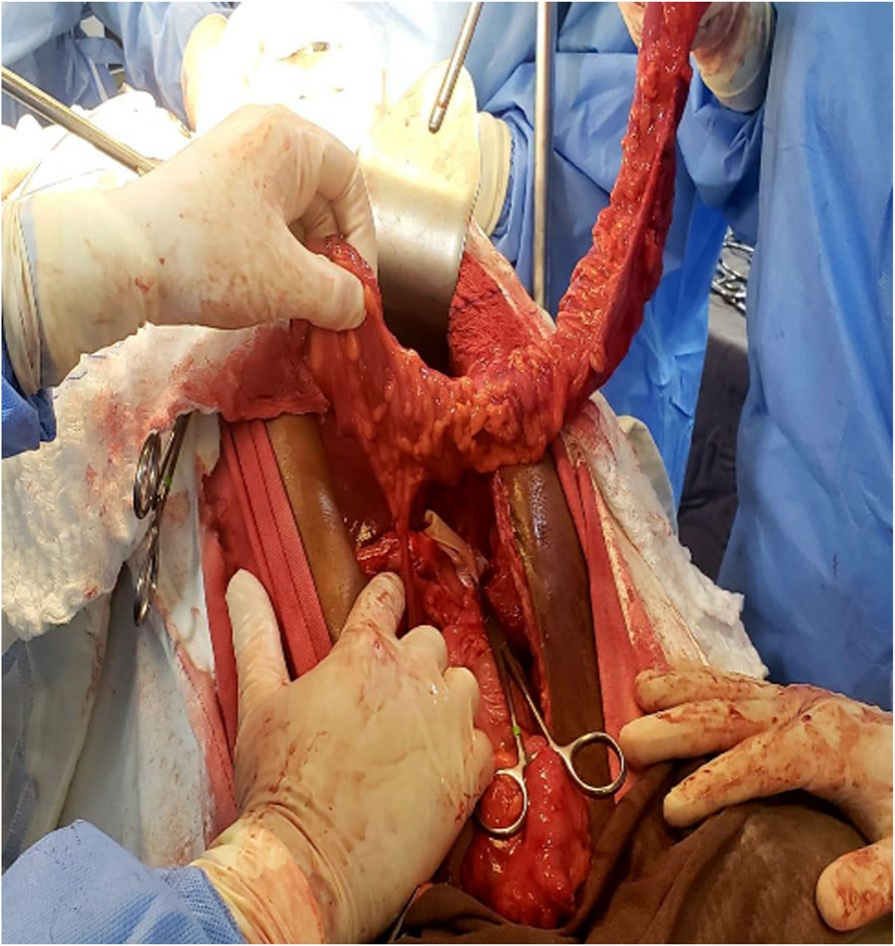
Colonic loop with right colic vessels

We started esophagocoloplasty with simultaneous cervicotomy and laparotomy, with the patient in the dorsal horizontal position under general anesthesia. We prepared the colon by dissecting its ligaments and testing perfusion by clamping the middle colic artery. Once adequate perfusion through the arc of Riolan was confirmed, we ligated the middle colic artery and transected the ascending and transverse colon using a linear stapler, preserving perfusion via the right colic vessels (Figs. 7 and Figs. 8).

We dissected the cervical esophagus as described above but did not perform esophagectomy, leaving it in the posterior mediastinum without transit (colonic bypass). We manually advanced the colon to the cervical region through a retrosternal tunnel. We performed the esophagocolonic anastomosis in the cervical region and the colonic anastomoses (ascending to transverse colon and gastrocolonic) in the abdomen, all in two layers.

### Study variables

We defined esophageal stricture based on the following: (1) upper gastrointestinal tract radiography showing reduced esophageal caliber with upstream contrast retention and no passage to the stomach; (2) endoscopy revealing luminal narrowing precluding scope progression and/or impossibility of endoscopic dilation; and (3) clinical signs including > 10% weight loss over two months, continuous sialorrhea, and severe dysphagia preventing oral intake.

### Statistical analysis

We expressed age as mean, standard deviation, minimum, and maximum. We presented other qualitative variables as absolute numbers (n) and percentages (%). To analyze the relationship between two qualitative variables, we used the chi-square test or Fisher’s exact test when appropriate, depending on expected cell counts. Statistical significance was set at *p* < 0.05. We performed statistical analyses using SPSS version XX (IBM Corp.).

### Ethical aspects

We conducted this study in accordance with the Ethical Standards of Scientific Research Involving Human Subjects from the Brazilian Health Department and the Declaration of Helsinki. The study was approved by the Research Ethics Committee of the Universidade Federal de Minas Gerais (UFMG) under opinion number 4.331.767 (CAAE: 30,995,717.0.0000.5149), on October 9, 2020. The requirement for informed consent was waived due to the retrospective design and use of de-identified data.

## Results

The mean age of the participants was 43.4 years (SD 17.7), and among the 101 patients, 63 (62.4%) were male. After endoscopic evaluation in the acute phase using the Zargar classification, we classified 19 patients (18.9%) as grade I, 8 (7.9%) as grade IIa, 25 (24.8%) as grade IIb, 31 (30.7%) as grade IIIa, and 18 (17.8%) as grade IIIb.

Baseline characteristics of patients in the esophagogastroplasty and esophagocoloplasty groups were generally comparable (Table 2). The mean age was 39.9 ± 16.3 years and 41.5 ± 10.6 years, respectively. The proportion of male patients was slightly higher in the esophagogastroplasty group (52.6% vs. 33.3%). Regarding stricture severity, similar rates of Zargar grade IIIa and IIIb injuries were observed between groups. The mean time from caustic ingestion to surgery was approximately 7 months in both groups Tables 3, Tables 4, Tables 5, Tables 6.

**Table 2:** Baseline characteristics of surgical groups

Characteristic	Esophagogastroplasty (*n* = 19)	Esophagocoloplasty (*n* = 15)
Age, mean ± SD (years)	39.9 ± 16.3	41.5 ± 10.6
Male, n (%)	10 (52.6%)	5 (33.3%)
Zargar grade IIIa, n (%)	8 (42.1%)	6 (40%)
Zargar grade IIIb, n (%)	8 (42.1%)	5 (33.3%)

**Table 3:** Zargar classification and incidence of esophageal stricture Distribution of patients according to Zargar classification grades and the corresponding occurrence of chronic esophageal strictures. Higher grades were significantly associated with increased stricture formation

Zargar	Stenosis No	Stenosis Yes	Total
I	18 (94,7%)a	01 (05,3%)b	19 (100%)
IIa	07 (87,5%)a	01 (12,5%)b	08 (100%)
IIb	15 (60,0%)a	10 (40,0%)a	25 (100%)
IIIa	12 (38,7%)a	19 (61,3%)b	31 (100%)
IIIb	02 (11,1%)a	16 (88,9%)b	18 (100%)
Total	54 (53,5%)	47 (46,5%)	101 (100%)

*Chi-square (df = 4) = 32.85

*p* < 0.001.*

*Different letters indicate statistically significant differences between groups.*

**Table 4:** Correlation between feeding route and development of esophageal stenosis. Distribution of patients according to the initial nutritional support method (jejunostomy or nasoenteric tube) and its association with subsequent development of esophageal strictures

Food Route	No Stenosis	Yes Stenosis	Total
Jejunostomy	16 (29,6%)a	37 (78,7%)b	53 (52,5%)
Nosoenteric tube	1 (1,9%)a	0 (0,0%)a	1 (1,0%)
Without jejunostomy	37 (68,5%)a	10 (21,3%)b	47 (46,5%)
Total	54 (100%)	47 (100%)	101 (100%)

**Table 5:** Endoscopic classification of caustic injuries in patients undergoing surgical treatment. Distribution of patients who required surgery according to Zargar endoscopic classification, illustrating the severity of esophageal injuries in the surgical cohort

EGD/Classification	N	%
II b	7	20,6
III a	14	41,2
III b	13	38,2
Total	34	100,0

**Table 6:** Distribution of surgical techniques and type of anastomosis performed. Correlation between the surgical reconstruction technique (esophagogastroplasty or esophagocoloplasty) and the type of anastomosis used (single-layer or two-layer, continuous or interrupted)

Operative technique	Single-Layer continuous	Single-Layer interrupted	Two-Layer interrupted	Total
Esophagectomy + esophagogastroplasty	11 (57,9%)	5 (26,3%)	3 (15,8%)	19 (100%)
Esophagocoloplasty	1 (6,7%)	0 (0,0%)	14 (93,3%)	15 (100%)
Total	12 (35,3%)	5 (14,7%)	17 (50,0%)	34 (100%)

Regarding the feeding route, 53 patients underwent jejunostomy and 1 used a nasoenteric tube.

Esophagectomy with esophagogastroplasty was performed in 19 (55.9%) patients, and esophagocoloplasty in 15 (44.1%).

The surgical technique used was related to the type of postoperative complication in Table 7.

**Table 7:** Association between surgical technique and postoperative complications. Comparison of postoperative complication rates (such as fistula, stricture, and mortality) between esophagogastroplasty and esophagocoloplasty groups. *Chi-square (df* = *8)* = *39.24; p* = *0.058*

Complication	Esophagectomy + esophagogastroplasty	Esophagocoloplasty	Total
Abscess	0 (0.0%)	1 (6.7%)	1 (2.9%)
Anastomotic stenosis	1 (5.3%)	0 (0.0%)	1 (2.9%)
Anastomotic leak	4 (21.1%)	8 (53.3%)	12 (35.3%)
Recurrent laryngeal nerve injury	1 (5.3%)	0 (0.0%)	1 (2.9%)
No complications	11 (57.7%)	3 (20.0%)	14 (41.2%)
Death	0 (0.0%)	3 (20.0%)	3 (8.8%)
Chylothorax	1 (5.3%)	0 (0.0%)	1 (2.9%)
Pulmonary thromboembolism	1 (5.3%)	0 (0.0%)	1 (2.9%)
Total	19 (100%)	15 (100%)	34 (100%)

In the esophagocoloplasty group, the postoperative mortality rate was 20% (3 out of 15 patients), whereas no deaths occurred in the esophagogastroplasty group. The anastomotic leak rate was 53.3% in the esophagocoloplasty group and 21.1% in the esophagogastroplasty group (*p* = 0.058, trend toward significance). Regarding long-term functional outcomes, most patients resumed oral feeding, but 12 patients (35%) required at least one postoperative dilation session due to anastomotic strictures. Nutritional follow-up revealed satisfactory weight recovery in the majority of cases, although detailed nutritional metrics were variably documented.

Most patients resumed oral feeding within the first few postoperative months. Only one patient developed a postoperative anastomotic stricture, which was successfully treated with endoscopic dilation. Overall, nutritional recovery was considered satisfactory, although detailed quantitative nutritional parameters were not consistently available due to the retrospective nature of data collection Tables 8.

**Table 8:** Anastomotic technique and related complications (leak and stenosis). Distribution of postoperative anastomotic complications (leakage and stricture) according to the type of anastomotic technique used (single-layer continuous, single-layer interrupted, or two-layer)

	Anastomosis technique			
Type of complication	Single-Layer continuous	Single-Layer interrupted	Two-Layer interrupted	Total
Stenosis anastomosis	1 (8,3%)^a^	0 (0,0%)^a^	0 (0,0%)^a^	1 (2,9%)
Anastomotic leak	0 (0,0%)^a^	4 (80%)^b^	8 (47%)^b^	12 (35,3%)
Total (anastomosis)	12	5	17	34

ChiQuadrado (4 gl) = 12,93. valor *p* = 0,012

^*^Different letters indicate differences between the two groups

## Discussion

### Key findings

In this retrospective cohort, we analyzed 101 adult patients with esophageal lesions resulting from caustic soda ingestion. Among these, 47 developed strictures, and 34 required surgical reconstruction after failure of endoscopic dilation. Our findings suggest that esophagogastroplasty was associated with lower rates of major complications and no postoperative mortality, whereas esophagocoloplasty was linked to higher leak and mortality rates, although differences did not reach statistical significance.

The esophageal injuries were classified using the Zargar endoscopic system. Unlike the original Zargar study, which included various caustic agents, our cohort focused exclusively on caustic soda ingestion, providing a more homogeneous population [13]. In our study, 46.5% of patients developed strictures. Specifically, stricture rates were 40% in grade IIb, 61.3% in grade IIIa, and 89.9% in grade IIIb (*p* = 0.000). This suggests that the severity of mucosal injury is a strong predictor of stricture formation, consistent with prior studies [4].

In Zargar’s study, only 38.3% of patients with esophageal injury developed strictures [13]. Mahawongkajit et al. reported 61.3% and 83.3% stricture rates for grades IIb and IIIa, respectively [10]. Hollenbach et al. observed that among patients who ingested alkaline substances, 44% were classified as IIIb, but they did not find a clear relationship between mucosal injury severity and stricture or mortality rates [4]. Our findings reinforce the importance of early endoscopic grading as a prognostic tool but also suggest that the type of caustic agent and individual tissue response might influence outcomes.

Cowan et al. analyzed 89 admissions due to caustic ingestion and found that among 29 patients undergoing EGD, 86.2% had Zargar grade ≥ IIb, supporting the high risk of severe strictures in higher grades [14].

Regarding nutritional support, 78.7% of patients who underwent jejunostomy developed strictures (*p* = 0.000), requiring either endoscopic or surgical intervention. Importantly, there was no mortality in this group. Surgical treatment was indicated for strictures refractory to dilation or for complete obstructions. While some authors suggest multiple dilation attempts before surgery, repeated dilations can increase perforation risk and worsen nutritional status, delaying necessary surgical intervention and increasing postoperative morbidity [7, 15].

There is limited data describing endoscopic classification in patients undergoing late-phase surgery. In our cohort, most surgical patients had higher Zargar grades, emphasizing the prognostic value of early endoscopic evaluation in anticipating future surgical needs.

All anastomoses in this study were performed manually. In the esophagogastroplasty group, 57.9% had single-layer continuous sutures, 26.3% had single-layer interrupted sutures, and 15.8% had two-layer sutures. In the esophagocoloplasty group, 93.3% underwent two-layer anastomoses. Literature comparisons show varied complication rates but often do not detail suture technique [8, 16–18]. Our data suggest that single-layer continuous anastomosis was associated with a lower fistula rate (*p* = 0.012), supporting its potential advantage over interrupted or two-layer techniques.

In our cohort, postoperative mortality was 20% in the esophagocoloplasty group (3 out of 15), while no deaths occurred in the esophagogastroplasty group. The leak rates were 53.3% in esophagocoloplasty and 21.1% in esophagogastroplasty (*p* = 0.058), indicating a trend but not statistical significance. Only one patient developed a postoperative anastomotic stricture requiring dilation. Overall, most patients successfully resumed oral feeding, indicating satisfactory functional recovery.

Potential confounding factors include surgeon experience, changes in perioperative management over time, and intraoperative decision-making based on gastric viability. The choice of technique was partially dependent on intraoperative findings and surgeon preference, introducing selection bias. The retrospective, single-center design further limits external validity and generalizability.

Previous studies reported diverse complication rates. Gupta described chylothorax after transhiatal esophagectomy requiring thoracotomy [19]. Teixidó et al. reported a 5% incidence of recurrent laryngeal nerve injury and 45% incidence of respiratory failure in patients undergoing esophagogastroplasty or esophagocoloplasty; the authors could not exclude pulmonary thromboembolism as a contributing factor [20]. Boukerrouche et al. observed a 2.8% mortality rate in esophagocoloplasty patients, with deaths attributed to pulmonary embolism and abdominal sepsis [21]. Benerjee reported a 10% rate of recurrent laryngeal nerve injury after esophagocoloplasty, which improved spontaneously after six weeks [17]. Our complication types are comparable to those described in the literature, though their incidence varies, likely reflecting heterogeneity in patient populations and surgical techniques.

In our series of chronic-phase surgeries, the overall incidence of anastomotic stenosis was 2.9%, and the fistula rate was 35.3%. Among patients who had single continuous plane anastomosis, there were no fistulas and 8.3% developed stenosis. In single interrupted plane anastomosis, 80% had fistulas and no stenosis. In two-layer anastomosis, 47% had fistulas and no stenosis. These findings suggest that single continuous sutures may reduce fistula risk (*p* = 0.012), which is consistent with observations in previous studies, despite small sample sizes and lack of statistical power.

Regarding functional outcomes, most patients resumed oral feeding in the first few postoperative months, and only one patient required postoperative endoscopic dilation for a stricture. Although overall nutritional recovery appeared satisfactory, detailed quantitative assessments (such as nutritional markers or standardized dysphagia scores) were not consistently available, representing an important limitation.

### Study limitations

This study has several limitations, including its retrospective design, small sample size, absence of a control group, and lack of long-term standardized functional outcome measures. The potential influence of unmeasured confounding factors (e.g., surgeon experience, evolving perioperative care protocols) further restricts the ability to draw definitive causal conclusions. Despite these limitations, our data provide valuable insights into the comparative safety and outcomes of esophagogastroplasty and esophagocoloplasty in chronic caustic esophageal strictures.

Future studies should focus on multicenter collaborations and standardized protocols to validate these findings, optimize surgical decision-making, and improve long-term patient outcomes in this challenging clinical context.

## Conclusion

Our data showed that endoscopic Zargar classification in patients who ingested caustic soda was a predictor of esophageal stricture.

The main indication for jejunostomy was the Zargar IIb classification, due to the risk of stenosis and food intolerance during follow-up, only 3.72% had complications with jejunostomy.

Between esophagectomy with esophagogastroplasty and esophagocoloplasty, the results imply a lower rate of complications on the first procedure, mainly due to mortality. Regarding the complications of the anastomoses, the single continuous plane suture technique showed a lower rate of fistula when compared to the others.

Publications dealing with the approach of patients with injuries secondary to the ingestion of caustic agents, both in the acute and chronic phases, are scarce. We believe it is imperative to carry out more studies in the area, so that the best treatments are offered to patients, always aiming at better clinical outcomes.

## Data Availability

The datasets generated and/or analyzed during the current study are not publicly available due to institutional privacy policies but are available from the corresponding author on reasonable request.
